# The Biological Roles of Class II Hydrophobins at the Cell Wall of Filamentous Ascomycetes

**DOI:** 10.1002/jobm.70205

**Published:** 2026-09-29

**Authors:** Wilhelm Hansberg, Omar Páez

**Affiliations:** ^1^ Instituto de Fisiología Celular Universidad Nacional Autónoma de México—UNAM Ciudad de México México; ^2^ Facultad de Ciencias Universidad Nacional Autónoma de México—UNAM Ciudad de México México

**Keywords:** ascomycete, cell wall, development, hydrophobins, surface

## Abstract

One of the most elusive aspects of fungal cell wall biology has been dilucidating the specific functions of most cell wall proteins. Class II hydrophobins are small amphipathic proteins produced exclusively by filamentous ascomycetes. A comparative synthesis of their biological functions across species has been lacking for this specific group of proteins. This review examines the available genetic, cell biological, and biochemical evidence on Class II hydrophobins in filamentous ascomycetes, with emphasis on Sordariomycetes, organized by both gene copy number and functional category. Gene deletion studies across species consistently reveal phenotypic effects on conidiation, desiccation resistance, host adhesion, and hyphal architecture, with severity scaling inversely with gene copy number. The evidence points to Class II hydrophobins acting as structural components integrated into or closely associated with the cell wall matrix, modulating its hydrophobic properties and mechanical characteristics. Abolishing these cell wall modulating properties can result in phenotypes related to fungal development and fitness. Understanding the contributions of Class II hydrophobins and other similar proteins to cell wall biology is directly relevant to fungal pathogenicity, host recognition, and antifungal strategies targeting the wall surface.

**Abbreviations**
CFTH1: hydrophobin 1 from Cladosporium fulvum
CmHYD1: hydrophobin 1 from *Cordyceps militaris*
CP: ceratoplatanin
CU: hydrophobin ceratoulmin from *Ophiostoma ulmi* or *O. novo‐ulmi*
EPL1: name of the best studied ceratoplatanin in *Trichoderma harzianum*
Fghyd2: hydrophobin 2 from *Fusarium graminearum*
Fghyd3: hydrophobin 3 from *Fusarium graminearum*
HFB10: hydrophobin 10 from *Trichoderma harzianum* and *T. guizhouense*
HFB4: hydrophobin 4 from *Trichoderma harzianum* and *T. guizhouense*
HFBI: hydrophobin 1 from *Trichoderma reesei*
HFBII: hydrophobin 1 from *Trichoderma reesei*
HFSs: hyphosphere secreted proteins
HYD2: hydrophobin 2 from *Metarhizium brunneum*
Hyd3: hydrophobin 3 from *Metarhizium robertsii*
MHP1: hydrophobin protein from *Magnaporthe oryzae*
MPG1: hydrophobin coded by the pathogenicity gene 1 from *Magnaporthe oryzae*
NC2: hydrophobin 2 from *Neurospora crassa*
PET: polyethylene terephthalate
RodA: rodlet hydrophobin A from *Aspergillus fumigatus*
SC3: hydrophobin 3 from *Schizophyllum commune*
SSCP: small secreted cysteine rich protein
UV: ultraviolet radiation

## 1 Introduction

Hydrophobins are small amphipathic proteins abundantly expressed in filamentous fungi and found across Ascomycota and Basidiomycota, with most fungal species possessing multiple hydrophobin genes [1, 2]. Hydrophobins self‐assemble on surfaces and hydrophobic/hydrophilic interfaces. This property renders them attractive molecules because of their numerous applications as surface‐changing agents, immobilizers and emulsifiers [3, 4, 5]. Derived from this industrial interest, the scientific literature on hydrophobins is quite vast. The functional capacity of hydrophobins surges from their amphipathic nature and conserved structure: all hydrophobins possess eight cysteine residues forming four disulfide bonds, creating a small compact globular structure with distinct hydrophilic and hydrophobic surfaces that drive their migration to interfaces [6].

Hydrophobins are classified based on their physicochemical properties in vitro [7]: Class I hydrophobins form highly stable rodlet layers with amyloid‐like properties that are extraordinarily resistant to dissociation, remaining insoluble in organic solvents, detergents, and alkali. In stark contrast, the molecular assemblies formed by Class II hydrophobins are less stable and can be dissociated by detergents and organic solvents. Also, the clustering patterns of hydrophobic and hydrophilic residues in their sequences were originally deemed to be clearly distinct. Taxonomically, Class I hydrophobins occur in both Ascomycota and Basidiomycota, whereas Class II hydrophobins are found exclusively in ascomycetes [7].

Frequently, no functional distinction is made between hydrophobin classes in the literature when their biological functions are discussed [8]. It is often stated that the main role in vivo of hydrophobins is conferring hydrophobicity to the aerial structures of fungi. The rodlet structures formed by assembled Class I hydrophobins are responsible for the water‐repellency of aerial mycelia and spores. This has been demonstrated for hydrophobin SC3 mutants in *Schizophyllum commune* that display wettable colony surfaces with defects in aerial hyphae formation [9], Mpg1 mutants from *Magnaporthe oryzae* exhibiting reduced rodlet production and impaired assembly [10] and the deletion of rodA from *Aspergillus fumigatus* that abolishes the rodlet layer on conidia and converts them from hydrophobic to hydrophilic [11]. The loss of hydrophobicity renders asexual spores unable to be dispersed by air currents.

Another frequently repeated role of hydrophobins is allowing aerial hyphae to emerge from an aqueous environment. This was based in a study from the Wessels laboratory showing that without the Class I hydrophobin SC3, aerial hyphae of *S. commune* were unable to emerge towards the air, out of the liquid culture [9]. Originally, this was attributed to the self‐attachment of secreted hydrophobins at the air‐water interface to aerial hyphae. Later, it was demonstrated that the ability of SC3 to render hyphae able to emerge out of the liquid is a consequence of its deposition on the hyphal surface directly as it is being expressed, not the consequence of its spontaneous attachment to hyphae at the medium/air interface [12]. Subsequent studies repeatedly state that hydrophobins allow aerial hyphae to breach the aqueous/air interface and grow into the air. However, this function in the emergence of aerial hyphae is true only for a small number of cases, such as for HFBI of *Trichoderma reesei* [13] and for Fghyd2 and Fghyd3 of *Fusarium graminearum* [14].

The importance of Class I hydrophobins is manifested beyond the water repellent properties of a rodlet coat. For example, the mutant of MPG1 from *M. oryzae* is unable to properly elaborate appressoria indicating that this hydrophobin is essential for adhesion, tissue penetration and full virulence in this species, with mutants showing diminished infection efficiency on the leaves of the host [10]. RodA also masks immunogenic cell wall components in *A. fumigatus* conidia, preventing cytokine production and neutrophil recruitment in infected host lungs [15]. Other Class I hydrophobins participate in the establishment of plant‐fungi interactions [16]. These examples of Class I hydrophobins illustrate that while rodlet formation and surface hydrophobicity represent the main function, they also participate in developmental transitions, and in both the interactions between fungi and their hosts and between fungi and their environment.

Class II hydrophobins do not self‐assemble into surface rodlets. This makes the mutant phenotypes in this class harder to be directly explained by a structure‐effect relationship. The differences between the two classes go beyond rodlet formation (Table 1). Since several in vitro studies of purified Class II hydrophobins have shown a high interfacial activity, it is frequently assumed that their function must depend on their status as secreted proteins, beyond the cell wall. A functional analysis of these proteins is often problematic because many ascomycete species possess multiple Class II hydrophobin genes: ranging from one to over ten in *Trichoderma* species [19]. The numerical dominance of Class II genes in Ascomycete genomes indicates that these hydrophobins play significant biological roles that remain to be elucidated. This review aims to set the basis for understanding the biological functions of this class of hydrophobins. From the analysis of mutant phenotypes in multiple species, this group of proteins fulfills important roles in cell wall based asexual morphogenesis and cell wall integrity, beyond conferring surface hydrophobicity.

**TABLE 1:** The contrasting properties of Class I and Class II hydrophobins.

	Class I	Class II
Fungal phyla	Present in both Ascomycota and Basidiomycota.	Exclusively found in the Ascomycota.
Structure variation [2, 17]	Larger sequence variation. Further divided in subclass IA (Ascomycota), IB (Basidiomycota) plus a not well defined subclass ‘mixed’ class I present in both phyla.	Higher sequence conservation and closer structural grouping.
Hydrophobic patch [2]	Larger patch exposed on the surface.	Amino acid residues in the hydrophobic patch highly conserved.
Supramolecular arrangement	Rigid rodlets	Elastic films
Stability of aggregates in vitro [18]	Dissolved by acid and base only. Resistant to ethanol and detergent.	Resistant to acid and base dissolution. Dissolved by ethanol and detergent.

## 2 The Structural Distinctiveness of Class II Hydrophobins

Within Class I hydrophobins there is a great amount of structural complexity. Hydrophobins identified in *Trichoderma* species were shown to form two well‐separated clades within Class I in ascomycetes, deviating in hydropathy, cysteine spacing, and protein surface patterns [20]. Intermediate forms emerged in *Aspergillus* and other genera that do not fit neatly into either class, exhibiting unique cysteine spacing and hydropathy profiles that led to proposals for an “intermediate hydrophobin class” [21, 22]. Phylogenetic studies subsequently identified multiple subdivisions within Class I with distinct clades (IB for those in the Basidiomycota and IA for those in the Ascomycota species), with principal component analysis demonstrating highly dispersed distribution of Class I sequences [17].

Unlike Class I, Class II hydrophobins exhibit remarkable stability in their classification. Principal component analysis shows tight clustering of Class II sequences [17]. This evolutionary stability indicates that Class II hydrophobins represent a distinct, coherent protein family, in contrast to Class I. Class II hydrophobins also maintain a highly conserved pattern of cysteine spacing and a consistent hydropathy distribution [22]. Sequence conservation patterns distinguish the two classes. Class I hydrophobins exhibit low conservation outside the eight‐cysteine framework, making automated classification difficult, whereas Class II hydrophobins show higher overall sequence conservation, enabling more reliable identification [22].

## 3 The Special Physicochemical Properties of This Class

The physicochemical properties of Class II hydrophobins have been characterized primarily through studies of purified proteins, with HFBI and HFBII from *T. reesei* serving as the primary model. These hydrophobins form non‐fibrillar, two‐dimensional crystalline films of interconnected mesh‐like protein networks, characterized by regular hexagonal lattices forming monolayers [23]. The assembly process of HFBI and HFBII at a high concentration, exhibits a stable tetrameric supramolecular form in aqueous solution, while lower concentrations yields a monomer‐dimer equilibrium [23, 24]. HFBI and HFBII differ in molecular packing: HFBI forms small single‐crystalline domains, while HFBII covers surfaces more readily with amorphous inter‐domain regions [23]. Hence, Class II hydrophobins from the same species vary in the geometry of their packing and surface coverage. At interfaces, the assembly proceeds through trimeric ensembles that organize into hexameric structures composed of two identical trimers, generating the characteristic polycrystalline films [25] (Figure 1A).

**FIGURE 1:**
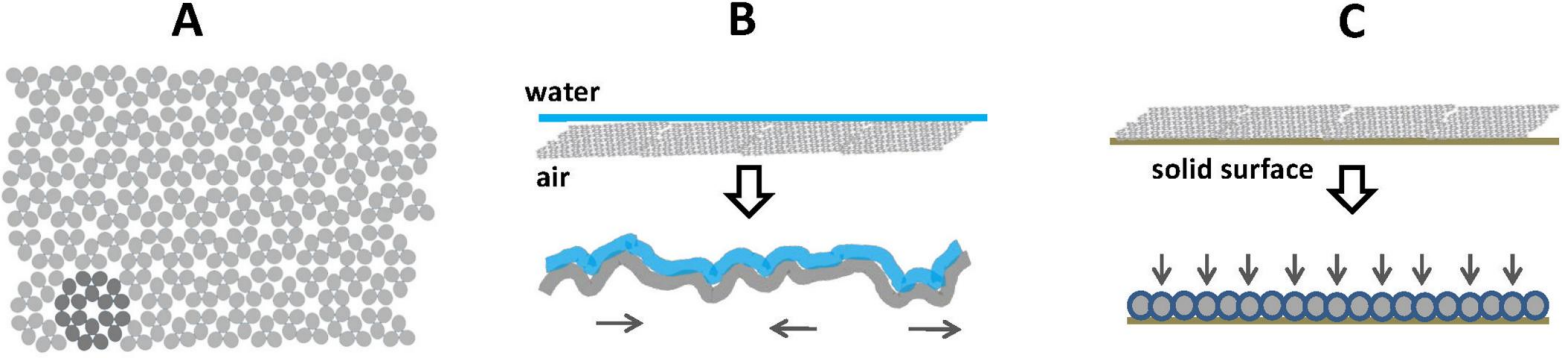
Structure of films and biological functions of Class II hydrophobins. (A) Films of Trichoderma reesei hydrophobins are formed from hexagonal lattices, one of which is highlighted in deep gray [25]. (B) These films assemble themselves at hydrophilic‐hydrophobic interfaces such as water‐air interfaces displaying remarkable elasticity [26]. (C) When attached on solid hydrophobic surfaces the films display a stronger force of interaction towards the material than the force of interaction between hydrophobin molecules. These films are stable under different environmental conditions such as PBS buffer (pH 7.4), acetic acid buffer (pH 5) and pure water [27].

In vitro, Class II films are disrupted by ethanol and detergents, whereas acid and base washes only partially disturb film morphology, without fully dissolving it [18, 28]. The fact that Class II films are prone to dissolution by detergents and ethanol has been highlighted in the literature but the fact that they mostly resist acid and base dissolution is seldom emphasized. At interfaces, Class II films exhibit exceptional mechanical properties. They deform the shape of water droplets and display a remarkable elasticity unparalleled by other surfactants [26] (Figure 1B). Underlying this behavior, it was found that the interaction force between a Class II hydrophobin protein and its hydrophobic substrate is superior than the interaction force between proteins [27]. This strong attachment to hydrophobic surfaces but weaker intermolecular association makes Class II films ordered and crystalline under appropriate conditions (e.g., a very hydrophobic substrate), yet responsive to solvent environment (Figure 1C).

Structural data from other Class II hydrophobins further illustrate the range of behaviors possible within this class. Ceratoulmin (CU) from *Ophiostoma ulmi* produces microstructures ranging from units to rods, fibrils, and finally membranes at air‐water interfaces [29], while CU from *Ophiostoma novo‐ulmi* forms films through a progressive hierarchical process beginning with pearl‐necklace‐like chains that collapse and merge into compact planar films [30]. NC2 from *Neurospora crassa* remains predominantly monomeric in solution, in stark contrast to the tetrameric behavior of HFBI and HFBII, yet forms networks of interconnected polygonal monolayers on hydrophobic surfaces with pores of 20–30 nm diameter [28]. Computationally, NC2 is predicted to possess higher surface activity than HFBII, and its films prove more resistant to shear stress and very susceptible to bending [31].

The Class II hydrophobin behaviors we have described come from in vitro studies where the proteins are acting alone on defined substrates. How these hydrophobins behave within the complexity of a fungal cell wall remains an open question. The variability in the number of Class II hydrophobin genes turns out to be informative: phenotypic severity following gene deletion tends to decrease with gene copy number.

## 4 From Multiple to One Class II Genes

The biological role of Class II hydrophobins has been examined in several ascomycete species. Only species belonging to the class Sordariomycetes are considered for these represent the majority of the published phenotypic evidence and constitute the best studied group in fungal cell biology. The following five ascomycete species contain both Class I and Class II hydrophobin genes in their genomes (Table 2). These species vary in their Class I gene repertoire, ranging from a single Class I gene in *M. grisea* (*oryzae*), to two Class I genes in *Cordyceps militaris* and in *Metarhizium brunneum*, to multiple Class I genes (> 4) in *F. graminearum*. Because contributions of Class II hydrophobins may be masked or compensated by the presence of Class I hydrophobins, in the following section, phenotypes are discussed first from species containing both Class I and Class II genes, and then in species containing only Class II genes, either one or multiple.

**TABLE 2:** Mutant phenotypes upon deletion of Class II hydrophobins in Sordariomycetes with genomes containing both Class I and Class II genes.

Number of class I genes in genome	4 (Fghyd1, Fghyd2, Fghyd3, Fghyd4)	2 (CmHYD3, CmHYD4)	2 (HYD1, HYD3)	1 (Mpg1)	1 (Mpg1)
Species	*Fusarium graminearum*	*Cordyceps militaris*	*Metarhizium brunneum*	*Magnaporthe oryzae*	*Magnaporte grisea (oryzae)*
Class II genes mutated	FgHyd5	CmHYD1,	HYD2	Mhp1 (kockdown by siRNA)	MHP1
Effect on hydrophobicity	Droplet angle diminished only.	Droplet angle diminished only.	ND	Wetted by water plus detergent.	Wetted by detergent and EDTA‐water.
Somatic growth	ND^a^	Diminished radial growth reported but effect is marginal.	ND	ND	ND
Formation of aerial structures	—	Reported as diminished by visual inspection only.	—	—	—
Conidiation	Augmented. Conidia shorter.	Significantly diminished.	Significantly diminished.	—	Unaffected. Only detectable in older cultures.
Specialized developmental structures	No effect in perithecia formation.	Completely unable to develop fruiting bodies.	Appresoria developed normally.	Reduced appressoria formation.	Reduced appressoria formation.
Stress tolerance and survival	—	The ∆Cmhyd1 strain grew more slowly in the presence of stressors.	—	—	Viability of conidia decreases after incubation at 4°C.
Pathogenicity and fitness	—	Lowest insect infection rates and longest time required.	Diminished mortality on insect larvae by 30%.	Infection symptoms are delayed.	Strong reduction in disease severity although it does elicit plant defense.
Additional observations	Penetration of hyphae through air‐water interfaces. ND	ΔCmhyd1 had the lowest germination rates. Less pigmentation.	Germination unaffected. Altered mycelial clumping in liquid. Less pigmentation.	Germinated conidia were less adhesive to the substrate.	Decreased rate of germination, especially in old conidia.
Reference	[14, 32]	[33]	[34]	[35]	[36]

^a^ ND, No difference to WT.

Among the eight species possessing only Class II hydrophobin genes (Tables 3 and 4), three possess more than four (*Trichoderma hazianum, Trichoderma guizhouense* and *Verticillium dahliae*) and two possess three genes (*T. reesei* and *Clonostachys rosea*). Species possessing a single Class II hydrophobin gene include *Cryphonectria parasitica* and *O. novo‐ulmi* (Table 5). Except for the saprobic species *T. reesei*, all of these species are fungi specialized in the interactions with other organisms: Plant pathogens (*F. graminearum, M. grisea* (*oryzae*), *V. dahliae, C. parasitica* and *O. novo‐ulmi*), entomopathogenic fungi (C. *militaris* and *M. brunneum*) and mycoparasitic fungi (*T. harzianum*, *T. guizhouense* and *C. rosea*). Hydrophobins have been researched in these species precisely because they could be part of the molecular arsenal these fungi use in effecting their cell wall mediated interactions. The next three sections examine the phenotypic effects on surface hydrophobicity, on aerial growth and conidiation, and on cell wall stress and fitness. A pattern emerges suggesting specific functions of Class II hydrophobin films at ascomycete cell walls.

**TABLE 3:** Mutant phenotypes upon deletion of class II hydrophobins in Sordariomycetes with genomes containing multiple Class II genes.

Species	*Trichoderma harzianum*	*Trichoderma guizhouense*	*Verticillium dahliae*	*Verticillium dahliae*
Class II genes mutated	hfb4 and hfb10 (hfb10 NP)	hfb4 and hfb10 (hfb10 NP)	Vdh1	Vdh1, Vdh2 (Vdh4 and Vdh5 no mutant phenotype)
Effect on hydro‐phobicity	ND^a^	Droplet angle diminished.	WT strains are wettable, but VDH1 mutants are unwettable.	ND
Somatic growth	ND	Minimal increased growth.	ND	ND
Formation of aerial structures	ND	Diminished aerial mycelia.	ND	ND
Conidiation	Diminished in the double mutant hfb4; hfb10.	Diminished in the double mutant hfb4; hfb10.	—	ND
Specialized developmental structures	—	—	Diminished production of microsclerotia.	ND
Stress tolerance and survival	Less desiccation resistance only.	significantly less desiccation resistance and UV resistance; higher freeze spore resistance.	Viability of conidia is decreased after desiccation.	ND
Pathogenicity and fitness	The single deletion of either hfb4 or hfb10 significantly improved the air dispersal ability.	Deletion of each single gene causes loss of air dispersal ability (attributed to loss of hydrophobicity).	ND. Albeit with a consistent acceleration in initial symptoms.	Vdh2 mutant is diminished in initial plant colonization. Lower infection efficiency of ΔVDH4ΔVDH5 at later stages.
Reference	[37]	[37]	[38]	[39]

*Note:* Species with > 4 class II genes.

^a^ ND, No difference to WT.

**TABLE 4:** Mutant phenotypes upon deletion of Class II hydrophobins in Sordariomycetes with genomes containing multiple Class II genes.

Species	*Trichoderma reesei*	*Trichoderma reesei*	*Trichoderma reesei*	*Clonostachys rosea*
Class II genes mutated	hfb1	hfb2	Hfb3 (Trhfb3)	hyd1, hyd2, hyd3
Effect on hydrophobicity	hfb1 mycelium is wettable.	Mycelium also wettable.	Easily wettable mycelium.	ND, only the dm hyd1; hyd3 shows slight decrease in hydrophobicity index.
Somatic growth	Hyphal diameter diminished. Only in liquid culture it produces less biomass.	ND^a^	Very small decrease in radial growth.	hyd1 and hyd2 relatively increased growth.
Formation of aerial structures	hfb1 has a faster growth of aerial hyphae.	ND	ND	—
Conidiation	Decreased conidiation.	Decreased conidiation.	Decreased conidiation.	hyd1 and hyd2 augmented, conidia form clumps when harvested in the double mutant hyd1; hyd2.
Specialized developmental structures	—	—	—	ND in root colonization ability, so infections cushions inferred to be unaffected.
Stress tolerance and survival	Not tested.	—	ND	Increased growth rate under NaCl and cold stress.
Pathogenicity and fitness	Not tested.	—	—	The ΔHyd1, ΔHyd3, and ΔHyd1ΔHyd3 strains overgrew 3 species faster than the WT.
ADDITIONAL TESTS	Aerial hyphae cannot grow out of liquid in the mutant strain.	—	ND in germination.	No differences in protein secretion.
Reference	[13]	[13]	[40]	[41]

*Note:* Species with 3 Class II genes.

^a^ ND, No difference to WT.

**TABLE 5:** Mutant phenotypes upon deletion of Class II hydrophobins in Sordariomycetes with genomes containing one Class II gene only.

Species	*Cryphonectria parasitica*	*Cryphonectria parasitica*	*Ophiostoma novo‐ulmi*	*Ophiostoma ulmi*
Class II genes mutated	Cryparin	Cryparin (knockdown by posttranscriptional gene silencing)	Ceratoulmin (CU)	Ceratoulmin (CU) OVEREXPRESSION
Effect on hydro‐phobicity	Mycelium becomes hydrophilic, reportedly by the droplet test.	The knockout phenotype is like the phenotype obtained by gene silencing.	After application of a drop of water to the mycelial surface, the CU‐mutant is water soaked; in WT it spreads but did not absorb.	Hydrophobicity was assessed by polystyrene adherence. CU overexpressing mutants showed higher affinity.
Somatic growth	ND^a^	—	ND in radial colony growth. ND in dry mycelial weight.	—
Formation of aerial structures	ND	—	CU‐mutant produced sparse aerial mycelia compared to WT.	The overexpressing strain exhibited an abundance of aerial hyphae.
Conidiation	ND	—	Not tested (occurs after infection).	Not tested (occurs after infection).
Specialized developmental structures	Stromal pustules containing perithecia do not emerge through the bark of host.	—	—	—
Stress tolerance and survival	ND in viability nor resistance to UV irradiation.	—	ND in the virulence parameters of the CU‐ mutant.	Increases resistance to desiccation.
Pathogenicity and fitness	ND in virulence when inoculated into chestnut trees.	—	—	ND in pathogenicity. CU overexpressing strains showed increased adherence to the beetle vector.
Reference	[42]	[43]	[44]	[45]

^a^ ND, No difference to WT.

### 4.1 Class II Hydrophobins Films Substitute the Function of Rodlets Only in Some Species

The disruption of Class II hydrophobin genes in species with Class I rodlets do not generally result in the loss of surface hydrophobicity. In *F. graminearum* (FgHyd5) and *C. militaris* (CmHYD1), mutants showed diminished droplet contact angles but did not become water‐soakable [14, 32, 33]. In *M. grisea/oryzae*, disruption of MHP1 caused aerial hyphae and conidia to became wettable only in detergent and EDTA containing solutions, not in water alone [35, 36]. Deletion of Hyd3 in *M. robertsii* and HYD2 in *M. brunneum* produced no detectable change in surface hydrophobicity [34, 46]. These findings indicate that Class II hydrophobins can modulate surface properties in some species. In species with multiple Class II genes but no Class I rodlets, Class II can fulfill the role of water repellency in some instances. In *C. rosea*, which possesses three genes, the triple deletion of hyd1, hyd2, and hyd3 produced no change in hydrophobicity index either [41]. In *V. dahliae*, where wild‐type strains are wettable, deletion of VDH1 resulted in a reversed phenotype, rendering spores hydrophobic, providing a striking case of cell wall modulation [38]. In *T. harzianum*, deletion of hfb4 and hfb10 produced no hydrophobicity phenotype, while hfb4 deleted in *T. guizhouense* resulted in diminished spore hydrophobicity [37]. In contrast, in *T. reesei*, which possesses three Class II genes, the individual deletion of each gene produced water‐wettable phenotypes [13, 40]. *T. reesei* provides the only example of multiple Class II hydrophobin films conferring surface hydrophobicity to hyphae.

In the two species with a single Class II gene, gene deletion resulted in a complete loss of water repellency. In *C. parasitica*, deletion of the hydrophobin cryparin resulted in mycelia becoming hydrophilic, a phenotype replicated by gene silencing [42, 43]. In *O. novo‐ulmi*, deletion of the ceratoulmin (CU) gene also produced water‐soakable mycelia, where water drops were absorbed rather than spreading across the surface as in wild‐type strains [44]. Overexpression of CU in the related species *O. ulmi* resulted in increased hydrophobicity as measured by polystyrene adherence assays [45]. Thus, Class II hydrophobins films can substitute the role of Class I hydrophobin rodlets in these two species.

### 4.2 Class II Hydrophobins Have Developmental Roles, Especially During Asexual Sporulation

A comparison of phenotypes reveals a role of Class II hydrophobins in different aspects of hyphal development and sporulation. The *C militaris* strain ΔCmHYD1 showed marginal growth defects [33] and the *M. brunneum* mutant ΔHYD2 showed an increased growth albeit not statistically significant difference to the WT strain [34]. Although aerial hyphae formation was reported as affected in Cordyceps [33] the difference seems too minimal to be considered biologically relevant. It is in asexual sporulation where important phenotypes were observed: conidiation was notably reduced in both *C militaris* and *M. brunneum* [33, 34], and *F. graminearum* ΔFgHyd5 mutants showed augmented sporulation with overall shorter conidia [32]. *C. militaris* mutants were completely unable to develop fruiting bodies [33] and *Magnaporthe* mutants displayed reduced appressorium formation [35, 36]. These defects in conidiation are suggestive of a morphogenetic role for Class II members, independent of conveying water repellency.

In species with multiple Class II genes but no rodlets, the importance in development is also evident. In *T. guizhouense*, deletion of hfb4 and hfb10 resulted in diminished aerial mycelium and conidiation [37]. In *T. harzianum*, the single deletion of the same genes produced no growth or aerial hyphae phenotypes, and the double mutant exhibited decreased conidiation [37]. In *V. dahliae*, deletion of Vdh1 resulted in diminished microsclerotia production but no changes in somatic growth or conidiation [38].

In *T. reesei*, all three Class II hydrophobins contribute to conidiation: hfb3 showed the most severe reduction and also produces less biomass in liquid cultures, while hfb1 additionally exhibited diminished hyphal diameter and faster aerial hyphae growth [13, 40]. In *C. rosea*, deletion of hyd1 or hyd2 resulted in a relative increase in somatic growth with hyd2 showing an augmented ability for root colonization [41]. The developmental alterations reported for Class II hydrophobin mutants are more remarkable in this group of fungi than in those bearing both classes in their genomes. The changes in the production of aerial mycelia and sporulation are indicative of a morphogenetic role for Class II hydrophobins. The observed changes in hyphal diameter and in *T. reesei* and the reduced conidiation in all *Trichoderma* species speak for a developmental role for Class II hydrophobins at the cell wall of aerial structures.

The mutant phenotypes in species containing a single Class II gene further demonstrate the developmental role of these hydrophobins. In these species, single gene knockouts produced minimal effects on somatic growth and conidiation but revealed an impact on aerial hyphae formation and specialized developmental structures. In *C. parasitica*, deletion of cryparin produced no changes in aerial hyphae, or conidiation, but stroma pustules containing perithecia failed to emerge through the bark of the host [42, 43]. This is a striking example of an alteration of the mechanical properties of hyphae. In *O. novo‐ulmi*, deletion of the ceratoulmin gene resulted in sparse aerial mycelia [44]. Overexpression of ceratoulmin in *O. ulmi* produced the opposite effect, with an abundance of aerial hyphae [45].

### 4.3 Class II Hydrophobins Affect the Integrity of the Cell Wall and Fungal Fitness

Assessments of stress tolerance and pathogenicity reveals notorious effects caused by the absence of Class II hydrophobins. *Cordyceps militaris* ΔCmHYD1 mutants showed reduced growth under cell wall and oxidative stressors (Congo Red, SDS, hydrogen peroxide) [33], and display reduced efficiency of infection. In *Trichoderma harzianum*, the deletion of hfb4 or hfb10 resulted in reduced desiccation resistance [37] and in *T. guizhouense*, deletion of the equivalent genes produced significantly reduced desiccation and UV resistance but increased freeze tolerance [37]. These results imply that Class II hydrophobins are paramount for the integrity of the cell wall in conidia. Deletion of *Clonostachys rosea* hyd1, hyd2, or hyd3 resulted in increased growth rates under both NaCl and cold stresses [41]. The overexpression of ceratoulmin in *O. ulmi* increased the resistance of conidia to desiccation [45]. The increased resistance to stress in these two species shows that Class II deletions may confer competitive advantages. The cell wall architecture in these mutants could be altered in ways that favor rapid growth or resource allocation and suggest that Class II hydrophobins may have important roles in the structuration of the cell wall.

Differences in spore viability can also be interpreted as a cell wall less resistant to stress or a more permeable cell wall. In this regard, in *Magnaporthe oryzae*, disruption of MHP1 resulted in a decreased conidial viability at 4°C and strong reductions in disease severity, with decreased germination rates particularly evident in older conidia [35, 36]. Furthermore, in *Verticillium dahliae*, deletion of Vdh1 decreased conidial viability under desiccation [38]. These observations reinforce the notion that Class II hydrophobins are involved in fungal cell wall fitness.

The interaction of fungi with their hosts is yet another manifestation of altered cell walls. *Metarhizium brunneum* ΔHYD2 mutants showed a 30% reduction in insect larval mortality, though germination remained unaffected [34]. *Verticillium dahliae* Vdh2 affected plant colonization, while Vdh4 and Vdh5 produced no observable mutant phenotypes [39]. The overexpression of ceratoulmin in *O. ulmi* resulted in an enhanced adherence to its vector beetle, though pathogenicity remained unaltered [45]. Ceratoulmin is recognized as a fitness factor for pathogenicity because of its role in turning the spore surface apt for adhesion. *Clonostachys rosea* deletion mutants of Class II hydrohobins exhibited enhanced mycoparasitic fitness, overgrowing competing fungi faster than wild‐type strains in confrontation assays [41] demonstrating that these cell wall proteins can affect fungal fitness. This effect of surface recognition by hyphae and conidia could be exerted by Class II hydrophobins either when secreted on the host or as a consequence of rearrangements at the cell wall.

Very few phenotypic studies support the notion that the function of Class II hydrophobins depends exclusively on their status as secreted proteins beyond the cell wall. For example, the effects of overexpression of ceratoulmin in *Ophiostoma ulmi* (increased hydrophobicity, aerial hyphae abundance, desiccation resistance, and vector adherence) in opposition to the effects upon gene deletion [45] can indeed be explained by quantitative contributions to the mycelial surface. Several other phenotypes reviewed here cannot simply be explained by a simple on/off switches for surface characteristics: The reversed hydrophobicity phenotype in *Verticillium dahliae*, where deletion of Vdh1 resulted in mutants becoming more hydrophobic than the wild‐type strain [38], indicates a direct modulation of cell wall properties rather than the simple switch of a hydrophobic coating. Class II hydrophobin deletion in *Trichoderma reesei* resulted in slimmer hyphae [13], indicating altered cell wall structure or thickness. Additionally, the *Cryphonectria parasitica* mutants [42], are a remarkable example of potentially altered mechanical properties of hyphae without disrupting the process of morphogenesis itself.

## 5 The Localization of Class II Hydrophobins In Vivo

Several studies have tracked the localization of Class II hydrophobins. HFBI of *Trichoderma reesei* was originally immunodetected as a secreted protein in the medium and also as part of the hyphal cell wall in SDS‐resistant complexes [47] and HFBII was also detected in the medium and on the spore cell wall, identified by protein sequencing [48]. Other early localization studies of Class II hydrophobins relied primarily on immunofluorescence approaches and pointed toward surface‐associated accumulation in specific developmental contexts: Cerato‐ulmin (CU) from *Ophiostoma* species was detected on the surface of conidia and germlings using an anti‐CU serum [49]. Similarly, CFTH1 from *Cladosporium fulvum* (Dothideomycetes) was localized by immunofluorescence to an extracellular matrix layer surrounding submerged hyphae in liquid cultures but also as part of the coating on the surface of aerial hyphae [50]. Notably, the aerial hyphae examined were surrounded only by air, not by liquid medium, suggesting that this secreted layer is a natural covering of the aerial wall.

Subsequent studies employing GFP fusion approaches have extended this picture. FgHyd5 in *Fusarium graminearum* displayed fluorescence exclusively in hyphae growing in direct contact with hydrophobic solid surfaces, Petri dishes or glass slides, while aerial mycelia not in contact with such materials showed no signal [32]. This contact‐dependent accumulation raises the possibility that FgHyd5 expression or deposition may be regulated by surface sensing. A contrasting result was obtained for TrHfb3 in *Trichoderma reesei*, where a GFP fusion construct yielded uniform signal in the cytoplasm of both conidia and hyphae, with no evident surface signal [40]. This result is difficult to interpret, as cytoplasmic GFP fluorescence may reflect a caveat of the fusion construct rather than genuine intracellular retention. Across these four studies, what is consistent is that Class II hydrophobins are associated with spores and aerial hyphae, placing them at precisely the surfaces most exposed to air and environmental challenge.

The most recent localization study examined HFB4 and HFB10 in *Trichoderma harzianum* and *T. guizhouense*, respectively, using fluorescent protein fusions [51]. Contrary to expectations, in vivo epifluorescence microscopy showed that during aerial hyphae development both proteins are accumulated massively at the intracellular region, stored predominantly in membrane‐bound vesicles and to a much lesser degree at a periplasmic cell wall location. Neither HFB4 nor HFB10 was associated with the cell wall in the manner previously observed for other Class II members. Both hydrophobins were detected extracellularly but with different patterns. HFB4 tends to accumulate intracellularly in conidiophores whereas secreted HFB10 showed greater affinity for structuration at the air‐water interface on the glass slide [51]. The authors held the view that the high intracellular expression observed reflects a function in vivo instead of representing incidental retention, and suggested that Class II hydrophobins may provide structural support for the erection of conidiophores by maintaining the turgor pressure in the aerial mycelium. Thus far, such an intracellular function has not been argued for other hydrophobins in other fungi.

## 6 Proteins Similar to Class II Hydrophobins

From all the reviewed studies, there are two species in which apparently no mutant phenotype was observed at all: *Metarrhizium robertsii* Δhyd3 mutants [46] and *Claviceps purpurea* cpphl [52]. It is possible that alterations at the cell‐wall level are not evident macroscopically but may be evidenced with a more detailed analysis of the cell wall. Also, it may be the case that the roles of Class II hydrophobins could be fulfilled by alternative wall proteins in certain fungi.

The cerato‐platanins (CPs) represent a well characterized family of secreted proteins with documented surface activity in filamentous ascomycetes. CPs are small (~ 12 kDa) secreted proteins defined by a conserved four‐cysteine pattern that forms two disulfide bonds [53, 54]. CPs share several functional properties with hydrophobins. Biochemically, CP family members self‐assemble into ordered aggregates and surface layers at hydrophobic/hydrophilic interfaces. These layers alter surface polarity and wettability in an opposite manner to hydrophobins and also bind chitin and *N*‐acetylglucosamine oligosaccharides, linking them directly to the fungal cell wall [54]. In *Trichoderma harzianum*, the cerato‐platanin EPL1 in involved in the mycoparasitic recognition of this fungus to their hosts, a biological function analogous to Class II hydrophobins [55].

Evidence for functional convergence between CPs and hydrophobins comes from a study by Zhao et al. [56]. Recombinant proteins from three distinct Small Secreted Cysteine rich Protein (SSCPs) families: hydrophobins, cerato‐platanins, and hyphosphere secreted proteins (HFSs) were assayed for surface‐conditioning activity on glass and polyethylene terephthalate (PET). All three families transformed initially hydrophobic abiotic surfaces toward a moderately hydrophilic state that promoted hyphal attachment [56]. This implies that the surface‐conditioning function performed by Class II hydrophobins could be partially fulfilled by CPs or HFSs. A functional substitution could also explain why some filamentous ascomycetes such as most *Aspergillus* species do not bear Class II hydrophobin genes in their genomes.

## 7 Concluding Remarks

The data reviewed here indicates that despite being water repellent in vitro, Class II hydrophobin films do not fulfill this role in many species. Such a role is restricted to some *Trichoderma* species and some species with a single class II gene. The abolishment of Class II hydrophobin films suggests a strong developmental role in species of filamentous ascomycetes. Class II films exert functions related to cell wall integrity that are suggestive of a role in the deposition of this structure. These films may integrate into existing cell wall matrices. The variable developmental and fitness phenotypes reviewed are likely consequences of an altered cell wall. Within the fungal cell wall, Class II hydrophobins would encounter polysaccharides, glycoproteins, and other wall components under spatial constraints very different from those of a free interface or a solid surface. Direct characterization of cell wall phenotypes caused by deletion of Class I and Class II hydrophobins in our research group has revealed important differences in polysaccharide arrangement and composition (manuscript in preparation). Understanding Class II hydrophobin contributions to the cell wall is directly relevant to fungal pathogenicity and host interactions, since the cell wall is the primary interface for recognition, adhesion, and defense responses in plant, insect, and mammalian hosts.

## Author Contributions

**Wilhelm Hansberg:** Funding acquisition, Resources, Validation, Writing – Review and Editing (supporting). **Omar Páez:** Conceptualization, Data curation, Formal analysis, Investigation, Methodology, Validation, Visualization, Writing – Original Draft Preparation, Writing – Review and Editing (lead).

## Data Availability

The data that support the findings of this study are available from the corresponding author upon reasonable request.
